# Draft genome sequence data of *Paenibacillus Polymyxa* strain TH2H2, isolated from a tomato flower in Korea

**DOI:** 10.1016/j.dib.2020.105824

**Published:** 2020-06-10

**Authors:** Gyeongjun Cho, Da-Ran Kim, Chang-Wook Jeon, Youn-Sig Kwak

**Affiliations:** a Division of Applied Life Science (BK21Plus), Gyeongsang National University, Jinju 52828, Republic of Korea; b Department of Plant Medicine and RILS, Gyeongsang National University, Jinju 52828, Republic of Korea

**Keywords:** Antibiotic genes, Biocontrol, *Paenibacillus polymyxa*, Phytobiome, Tomato

## Abstract

Members of the genus *Paenibacillus* are known for their production of useful substances, and some species of the genus are recognized to be plant growth-promoting rhizobacteria. *Paenibacillus polymyxa* TH2H2, isolated from a tomato flower, had antifungal activity. Here, the draft genome sequence of *Paenibacillus polymyxa* TH2H2 is reported. The 5,983,104-bp genome, with a G+C content of 45.31%, comprised 5,221 protein-coding genes, 64 ribosomal RNA and 100 transfer RNA. Three intact antibiotic biosynthesis gene clusters were identified using antiSMASH. These encoded the antifungal agent fusaricidin and two antibacterial agents, tridecaptin and polymyxin. Sequence data have been deposited in the DDBJ/ENA/GenBank database under the accession number RPDG01000000. The version described in this paper is RPDG00000000.1. The BioProject ID in the GenBank database is PRJNA505713.

Specifications table

**Table:** 

Subject	Microbiology
Specific subject area	Phytobiome and a keystone taxon
Type of data	Table, figure
How data were acquired	Genome sequencing with MiSeq paired-end protocol at Chunlab Inc. Republic of Korea
Data format	Raw and Analyzed
Parameters for data collection	Bacterial genomic DNA was extracted from a culture of *Paenibacillus polymyxa* TH2H2
Description of data collection	Genome features (Table 1), genome map (Fig. 1a), EggNOG functional category (Fig. 1b), genome annotation and COG (Supplementary data 1), secondary metabolite gene cluster (Supplementary data 2)
Data source location	*Paenibacillus polymyxa* TH2H2 was obtained from a healthy tomato flower in the city of Jinju, Republic of Korea (GPS: 35.2109N, 128.1164E)
Data accessibility	Repository name: DDBJ/ENA/GenBank Data identification number: RPDG01000000. The version described in this paper is RPDG00000000.1 Direct URL to data: https://www.ncbi.nlm.nih.gov/nuccore/RPDG00000000.1 The BioProject ID in GenBank is PRJNA505713 (https://www.ncbi.nlm.nih.gov/search/all/?term=PRJNA505713) The data processing R code is available in a GitHub repository (https://github.com/gyeongjunCho/TH2H2_draft_genome)
Related research article	Da-Ran Kim, Jun-Taek Lee, Hye sun Kim, Chang Wook Jeon, Youn-Sig Kwak. Selection of biocontrol agent of tomato gray mold disease from flower and pollinator hive. The Korean Journal of Pesticide Science 21 (2017) 90-96. https://doi.org/10.7585/kjps.2017.21.1.90

## Value of the data

• The complete genome sequence of *P. polymyxa* TH2H2 provides essential information about the strain that can be applied to plant protection research and biological control of plant.
• In the genome of *P. polymyxa* TH2H2, 37 antibiotic-related metabolite gene clusters were predicted, indicating that the strain could be valuable in investigations of plant-microbe interactions.
• *P. polymyxa* TH2H2 genome data provides information about species of the genus *Paenibacillus* that will be useful to the wider microbial research community.

## Data Description

Species of the genus *Paenibacillus* are rod-shaped, aerobic or facultatively anaerobic, endospore-forming bacteria with Gram-positive and Gram-variable attributes. They inhabit various sites including soil, sediment, sewage, caves, compost, water, plant and animal tissues, and so on. Some species of the genus *Paenibacillus* are reported to be plant growth-promoting rhizobacteria that fix nitrogen, solubilize phosphate and act as antagonistic agents for plant pathogens [1,2]. Members of the genus *Paenibacillus* produce a range of molecules such as amylases, cellulases, lipases, pectinases, oxygenases, dehydrogeneases, lignin-modifying enzyme, exopolysaccharides and antibiotic agents, and these products have applications in the food, paper, biofuel, textiles, agriculture, detergents and medicine industries [1]. *Paenibacillus polymyxa* TH2H2 was previously isolated from a tomato flower [3]. The strain had antifungal activity, accompanied by high levels of cellulase and proteinase activity, towards the tomato pathogen *Botrytis cinera* [3].

**Fig. 1:**
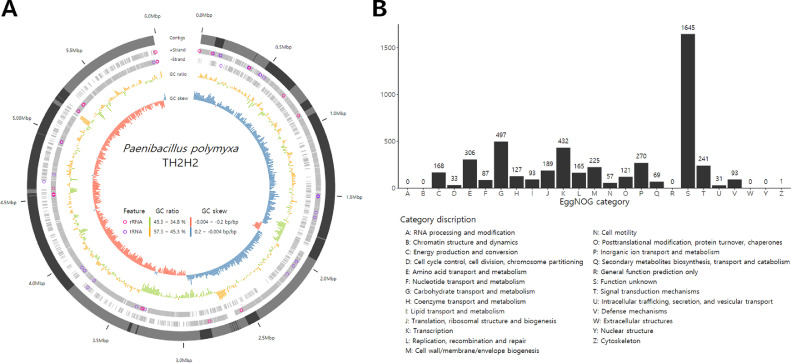
Pseudogenome map and Clusters of Orthologous Groups (COG) analysis of *P. polymyxa* TH2H2 coding sequences. (A) In the draft genome map, the gray outermost ring is segmented by contig length; light gray and dark gray refer to odd and even numbers of contigs, respectively. The next two gray rings indicate CDS in each DNA direction. The remaining bar graph rings are GC ratio and GC skew per 10,000 bp. (B) EggNOG functional category classification. The x-axis represents gene functional category and the y-axis represents the frequency of each functional category.

The *P. polymyxa* TH2H2 genome was sequenced to 5,983,104 bp, and comprised 113 contigs and a G+C content of 45.31% (Fig. 1A, Table 1, supplementary data 1). There were 5,221 coding sequences (CDS), 64 ribosomal RNA and 100 transfer RNA. Cluster of Orthologous Groups (COG) analysis categorized 4,646 genes (supplementary data 1). With the exception of genes with unknown functions, the most frequently observed category was carbohydrate transport and metabolism, followed by transcription, then energy production and conversion (Fig. 1B). Thirty-seven secondary metabolite gene clusters were predicted using antiSMASH (supplementary data 2). Among these, three complete non-ribosomal peptides (NRP) clusters were identified. The first NRP was fusaricidin biosynthetic gene cluster [4]; fusaricidin is a recognized antifungal agent. The other two NRPs were tridecaptin and polymyxin biosynthetic gene clusters [5,6], both of which are antibacterial agents targeting Gram-negative bacteria.

**Table 1:** Genome features of *Paenibacillus polymyxa* TH2H2.

Genome feature	Value
Genome size	5,983,104 bp
G+C content	45.31%
GC skew	-0.004 bp/bp
Number of rRNA genes	64
Number of tRNA genes	100
Number of ORFs	5,221
Number of contigs	113
Sequencing depth of coverage	139.97×
N50	466,974
L50	5

## Experimental Design, Materials and Methods

*P. polymyxa* TH2H2 was cultivated as previously described [3]. Genomic DNA was extracted using the CTAB method [7]. Illumina Miseq paired-end (2× 300 bp) sequencing of *P. polymyxa* TH2H2 was performed by Chunlab Inc. (Seoul, Korea). Briefly, genomic DNA was processed into a library using NEBNext dsDNA fragmantase (NEB, Hitchin, UK) and TruSeq RNA Library Prep Kit v2 (Illumina, Inc., San Diego, CA, USA). The sequencing generated 4,895,864 reads of 1,102,733,884 bp when adapter sequences were removed. The reads were assembled into 113 contigs of 137.97× coverage using SPAdes (version 3.10.1) [8]. The absence of sequence contamination was confirmed (supplementary Figure 1) using ContEst16 (https://www.ezbiocloud.net/tools/contest16s) [9]. COG analysis was conducted using the EggNOG database [10]. The above analysis procedures were automatically perfomed with CLgenomics (version 1.55). Secondary metabolite gene clusters were predicted using antiSMASH (version 4.2.0; https://antismash.secondarymetabolites.org) [11]. All data were graphed in R (version 3.6.3), and the R code used was shared on a GitHub repository (https://github.com/gyeongjunCho/TH2H2_draft_genome).

## Declaration of Competing Interest

The authors declare that they have no known competing financial interests or personal relationships which have, or could be perceived to have, influenced the work reported in this article.

## Appendix Supplementary materials

Image, application 1

Image, application 2

Image, application 3

## References

[1] Grady E.N., MacDonald J., Liu L., Richman A., Yuan Z.-C. (2016). Current knowledge and perspectives of *Paenibacillus*: a review. Microb. Cell Fact..

[2] Weselowski B. (2016). Isolation, identification and characterization of *Paenibacillus polymyxa* CR1 with potentials for biopesticide, biofertilization, biomass degradation and biofuel production. BMC Microbiol.

[3] Kim D.-R. (2017). Selection of biocontrol agent of tomato gray mold disease from flower and pollinator hive. Korean J. Pestic. Sci..

[4] Li J., Jensen S.E. (2008). Nonribosomal biosynthesis of fusaricidins by *Paenibacillus polymyxa* PKB1 involves direct activation of a d-amino acid. Chem. Biol..

[5] Lohans C.T. (2014). Biochemical, structural, and genetic characterization of tridecaptin A1, an antagonist of *Campylobacter jejuni*. ChemBiochem.

[6] Choi S.-K. (2009). Identification of a plymyxin synthetase gene cluster of *Paenibacillus polymyxa* and heterologous expression of the gene in *Bacillus subtilis*. J. Bacteriol..

[7] William S., Feil H., Copeland A. (2012). Bacterial genomic DNA isolation using CTAB. Sigma.

[8] Bankevich A. (2012). SPAdes: A new genome assembly algorithm and its applications to single-cell sequencing. J. Comput. Biol..

[9] Lee I. (2017). ContEst16S: an algorithm that identifies contaminated prokaryotic genomes using 16S RNA gene sequences. Int. J. Syst. Evol. Microbiol.

[10] Powell S. (2014). eggNOG v4.0: nested orthology inference across 3686 organisms. Nucleic Acids Res.

[11] Medema M.H. (2011). antiSMASH: rapid identification, annotation and analysis of secondary metabolite biosynthesis gene clusters in bacterial and fungal genome sequences. Nucleic Acids Res.

